# Supplementary material for: An Efficient Method for Vault Nanoparticle Conjugation with Finely Adjustable Amounts of Antibodies and Small Molecules
Source: Int J Mol Sci. 2024 Jun 16;25(12):6629. doi: 10.3390/ijms25126629 (PMC11203631; doi:10.3390/ijms25126629)
Supplement: Supplementary file 1 [file ijms-25-06629-s001.zip › ijms-3014206-supplementary.pdf]

Supplementary information

Assessing a dissociation constant of two molecular partners on the basis of the ratio bound/total

We have developed a mathematical model in view of assessing antibody affinity for vault-Z. As a premise, it is assumed that each MVP-Z subunit behaves as an independent entity. This is not necessarily true, but the results themselves will determine to what extent this assumption holds true, as documented below.

Let the antibody be represented as **A**, MVP-Z as **M** and the complex MVP-Z/antibody as **MA**; then, the following dissociation equilibrium applies:

$$\mathbf{MA \leftrightarrows M + A}$$

The related dissociation constant is:

$$K_D = \frac{[M]\,[A]}{[MA]}$$

Then, the following relationships can be introduced:

$$[M] + [MA] = [M]_t$$
$$[A] + [MA] = [A]_t$$

Which also equal:

$$[M] = [M]_t - [MA]$$
$$[A] = [A]_t - [MA]$$

where the subscript t indicates the total concentration (bound and free) of either molecular species. By replacing the latter equations in the dissociation constant formula, the following relationship is yielded:

$$K_D = \frac{([M]_t - [MA])\,([A]_t - [MA])}{[MA]}$$

The equation can be further rearranged as follows:

$$K_D = \frac{[M]_t\,[A]_t - [M]_t\,[MA] - [MA]\,[A]_t + [MA]^2}{[MA]}$$

Let be now introduced the quantity **S**, which is defined as follows:

$$S = \frac{[MA]}{[M]_t}$$

or:

$$[MA] = [M]_t\,S$$

So, **S** is the saturation fraction of the MVP-Z subunits by the antibody and has a theoretical range of 0 (no saturation) to 1 (100% saturation). By replacing **S** in the rearranged dissociation constant formula, it results the following:

$$K_D = \frac{[M]_t\,[A]_t - [M]_t^2\,S - [M]_t\,[A]_t\,S + [M]_t^2\,S^2}{[M]_t\,S}$$

By dividing numerator and denominator by $\mathbf{[M]_t\ S}$, the equation results in:

$$\mathbf{K_D = [A]_t\ S^{-1} - [M]_t - [A]_t + [M]_t\ S}$$

This represents a generally valid, quadratic saturation equation, i.e., without any constraint regarding the range of concentrations and molar ratios between the two molecular partners.

Based on this theoretical framework, the prerequisites for the $\mathbf{K_D}$ to be determined are: 1) knowing the exact total antibody ($\mathbf{[A]_t}$) and MVP-Z ($\mathbf{[M]_t}$) concentrations in the incubation mixture, as selected in the experimental design; 2) determining the **S** value for each incubation mixture. In our experimental system, concentrations of bound antibody (**[MA]**) and total MVP-Z ($\mathbf{[M]_t}$) are proportional to the respective amounts collected by ultracentrifugation of the incubation mixtures and assessed by either densitometry or mass spectrometry (Materials and Methods, Section 4.9). It should be also remarked that $\mathbf{K_D}$ measurements performed in this way offer the considerable advantage of being unaffected by the extent of pellet recovery, at least as far as the amount of recovered protein would be sufficient for reliable measurements. Indeed, whatever the protein loss may be, the obvious assumption is that all protein species present in the pelleted specimen would be recovered in equal proportions.

It should be however pointed out that this approach cannot provide reliable $\mathbf{K_D}$ assessments values when the affinity is very high and accordingly the ratio bound to total antibody exceed 95%, just because the differences detected in this way would lie in the range of the experimental error. Instead, this is feasible at lower values, so we could estimate apparent $\mathbf{K_D}$ values of 200 and 693 nM at molar ratios Tz/vault-Z of 20 and 40, respectively. This, in turn, corresponds to 70% and 41% of bound antibody, respectively.

Figures

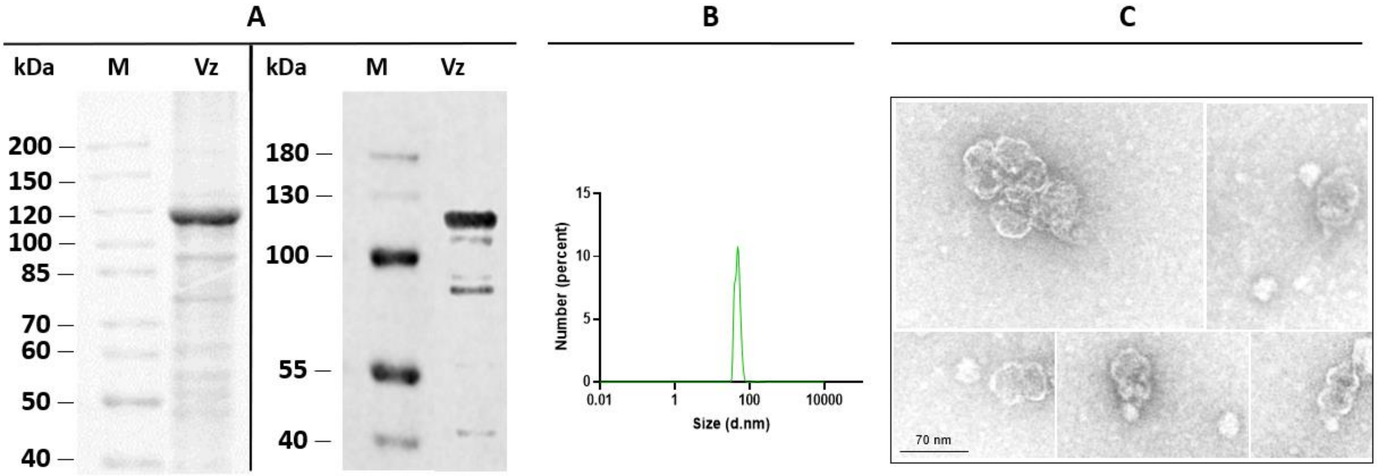

Figure S1. Characterization of vault-Z purity and identity by different analytic methods. A) SDS-PAGE (8% gel) of purified vault-Z. Left panel: 10 µg of purified protein (Vz) was loaded onto the gel and stained by Imperial Protein Stain (Thermo Scientific). Right panel: Western blot of the same sample. M: standard proteins with the respective molecular weights (kDa): PageRuler™ protein ladder (ThermoFischer) either unstained (left) or prestained (right). B) DLS analysis of purified vault-Z. Results are presented as number-weighted particle size distributions. Size: 52.27 ± 6.21 nm (major component: 99.4%); 191.00 ± 43.08 nm (minor component: 0.6%); polydispersity index (PDI): 0.272. Result quality: good. The profile represents the mean of three independent replicates. C) TEM images of vault-Z NPs stained by uranyl acetate. Light-colored objects are non-protein artifactual clumps. Other details are reported in the respective paragraphs of Materials and Methods, Sections 4.5 to 4.7.

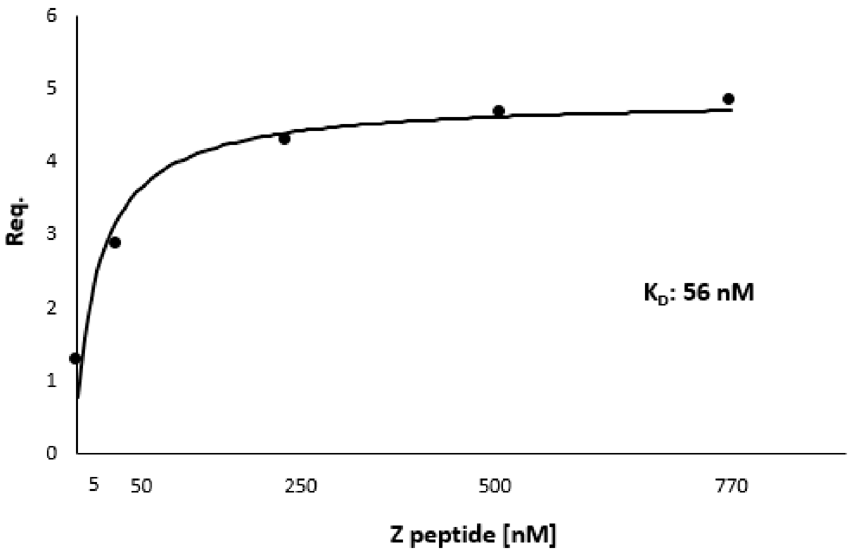

Figure S2. Plot Req/conc Z peptide from the set of SPR sensorgrams shown in Figure 3. Data fitted with affinity model by the software BiacoreX100 Evalutation 2.

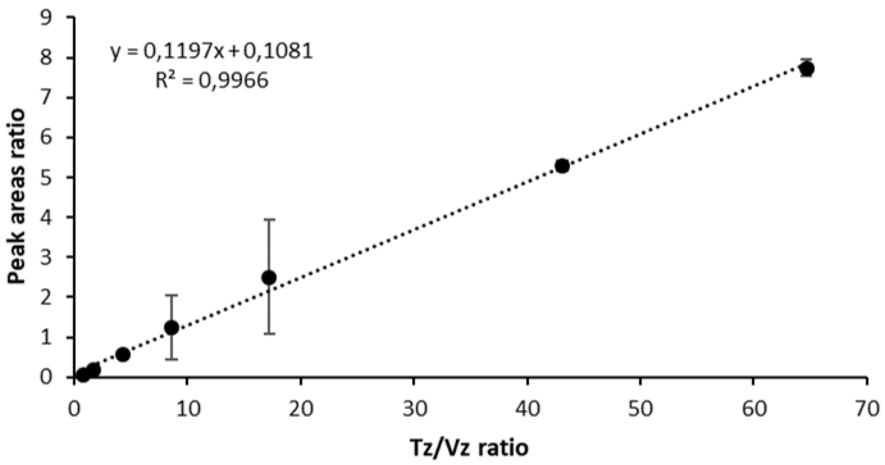

Figure S3. Calibration curve of Tz/vault-Z ratios by LC/MS. The curve is constructed by a linear interpolation of data points representing the chromatographic peak intensities of six peptides for both Tz and vault-Z proteins. The peptides are obtained by tryptic digestion of mixtures of the two proteins at defined molar ratios. Error bars represent standard deviations over three independent experiments.

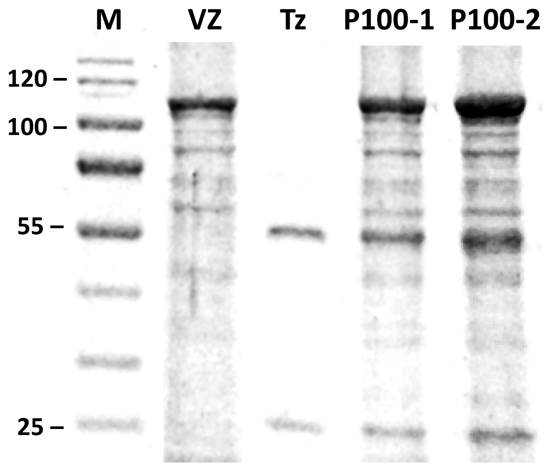

Figure S4. Tz retention by vault-Z (VZ) after two ultracentrifugations at 100,000 x g. Suitable amounts of vault-Z and Tz were mixed at a molar ratio 10:1, preincubated and ultracentrifuged at 100,000 x g. Then, the supernatant was discarded, the pellet resuspended in 7 mL and again spun down as above. The two pellets (100-1 and 100-2, respectively) were analyzed by SDS-PAGE (12% gel) along with marker proteins and VZ and Tz samples used as references. The ratios MVP-Z/Tz (heavy chain), as assessed densitometrically, were 0.72 (100-1) and 0.74 (100-2), indicating complete retention of the antibody. Other experimental details are reported in Section 4.9.

CTTGGCCATTGAAACGGAGGCT**GAGCTC**CAGAGGGTCCAGAAGGTCCGAGAGCTGGAACTGGTCTATGCCCG
GGCCCAGCTGGAGCTGGAGGTGAGCAAGGCTCAGCAGCTGGCTGAGGTGGAGGTGAAGAAGTTCAAGCAGAT
GACAGAGGCCATAGGCCCCAGCACCATCAGGGACCTTGCTGTGGCTGGGCCTGAGATGCAGGTAAAACTGCT
CCAGTCCCTGGGCCTGAAATCAACCCTCATCACCGATGGCTCCACTCCCATCAACCTCTTCAACACAGCCTT
TGGGCTGCTGGGGATGGGGCCCGAGGGTCAGCCCCTGGGCAGAAGGGTGGCCAGTGGGCCCAGCCCTGGGGA
GGGGATATCCCCCCAGTCTGCTCAGGCCCCTCAAGCTCCTGGAGACAACCACGTGGTGCCTGTACTGCGCTT
TAACATGCAGCAGCAGCGCCGCTTTTACGAGGCCCTGCACGACCCCAACCTGAACGAGGAGCAGCGCAACGC
CAAGATTAAGAGCATTCGCGACGAC**TAG**GGTACCTCGAGCCGCG**GCGGCCGC**CAGCTTTCTAGAACAAAAAC
TCA

Figure S5. The Z peptide-encoding nucleotide sequence (underlined). The sequence carries sticky ends complementary to those resulting from *Not*I/*Sac*I digestion sites of the pGAPZB plasmid (highlighted in blue and green, respectively) and exposing the MVP C-terminal sequence, which allowed in-frame fusion and plasmid re-circularization. The stop codon is in bold.
